# African Rice (*Oryza glaberrima* Steud.): Lost Crop of the Enslaved Africans Discovered in Suriname^1^

**DOI:** 10.1007/s12231-010-9111-6

**Published:** 2010-03-05

**Authors:** Tinde Van Andel

**Affiliations:** Netherlands Centre for Biodiversity, Section NHN, Leiden University, Leiden, the Netherlands

**Keywords:** Food offering, Maroons, *Oryza glaberrima*, Suriname, traditional agriculture

## Abstract

**African Rice (** ***Oryza glaberrima*** **Steud.): Lost Crop of the Enslaved Africans Discovered in Suriname**. African rice (*Oryza glaberrima* Steud.) was introduced to the Americas during the slave trade years and grown by enslaved Africans for decades before mechanical milling devices facilitated the shift towards Asian rice (*O. sativa* L.). Literature suggests that African rice is still grown in Guyana and French Guiana, but the most recent herbarium voucher dates from 1938. In this paper, evidence is presented that *O. glaberrima* is still grown by Saramaccan Maroons both for food and ritual uses. Saramaccan informants claim their forefathers collected their first “black rice” from a mysterious wild rice swamp and cultivated these seeds afterwards. Unmilled spikelets (grains with their husk still attached) are sold in small quantities for ancestor offerings, and even exported to the Netherlands to be used by Maroon immigrants. Little is known of the evolution of *O. glaberrima*, before and after domestication. Therefore, more research is needed on the different varieties of rice and other “lost crops” grown by these descendants of enslaved Africans who escaped from plantations in the 17th and 18th centuries and maintained much of their African cultural heritage in the deep rainforest.

## Résumé

**Afrikaanse rijst (** ***Oryza glaberrima*** **Steud.): Een verloren gewaand slavengewas ontdekt in Suriname.** Afrikaanse of zwarte rijst (*Oryza glaberrima* Steud.) is door slavenschepen meegenomen naar het Amerikaanse continent, alwaar het meer dan honderd jaar lang door slaven werd verbouwd totdat de uitvinding van mechanische pelmolens de overgang naar Aziatische rijst (*O. sativa* L.) inluidden. In de literatuur wordt gesuggereerd dat Afrikaanse rijst nog steeds wordt gecultiveerd in de Guianas, maar het meest recente herbariumexemplaar stamt uit 1938. In dit artikel wordt het bewijs geleverd dat *O. glaberrima* nog steeds wordt gekweekt, zowel voor voedsel als ritueel gebruik, door Saramaccaanse Marrons, afstammelingen van slaven die in de 17^e^ en 18^e^ eeuw de plantages ontvluchtten. Ongepelde rijstkorrels worden in kleine hoeveelheden verkocht voor voorouderrituelen, zowel op de markt in Paramaribo als in Surinaamse winkels in Nederland. Er is weinig bekend over de evolutie van *O. glaberrima* voor en na domesticatie. Meer onderzoek is daarom nodig naar de verschillende rijstvariëteiten en andere ‘vergeten gewassen’ van de Marrons, die in het diepe regenwoud van Suriname veel van hun Afrikaanse cultuur wisten te behouden.

## Introduction

Domesticated some 3,500 years ago from wild ancestors along the Niger River in Mali, African rice (*Oryza glaberrima* Steud.) was introduced to the New World in the 17th century by means of the slave trade. Unprocessed rice was purchased by slave traders in West Africa to serve as ship provision, and later grown by the enslaved in their home gardens (Carney 2001 and 2005). Until the first Asian rice (*O. sativa* L.) was introduced in the 1690s, the entire rice cultivation in South Carolina must have been based on *O. glaberrima* (Carney 2001; Salley 1919). The traditional farming skills of the displaced Africans played a crucial role in adapting the crop to different New World environments, as rice was the primary food staple in the countries where many of them were born. The introduction of mechanical hulling devices on the South Carolina rice plantations in the 18th century facilitated the shift towards Asian rice (Carney 2001 and 2005).

African rice always has short, rounded ligules, simply branched, erect panicles with small spikelets that have an olive or reddish–brown to black husk, and a bran color that shifts from reddish–brown to purple. Some varieties have a long, straight apical awn (Bezançon and Diallo 2006; Linares 2002). Because of its dark bran, *O. glaberrima* is often called “black” or “red rice.” African rice has many unique traits that make it a suitable crop for low–input, subsistence agriculture, such as a tolerance to salt, drought, flooding, pest–resistance, weed competitiveness, and the ability to grow on infertile, acid soils. *O. glaberrima* also matures faster than Asian varieties and its wide leaves shade out weeds (Harlan 1995; Linares 2002; Sarla and Mallikarjuna Swamy 2005). Negative features are a lower yield, seeds that scatter easily, and a notorious difficulty of milling. To avoid breakage of the grains, *O. glaberrima* must be milled by hand with a wooden mortar and pestle, after which the hulls must be removed through winnowing the cereal by hand (Carney 2001; Linares 2002).

Provided the environment was suitable for the crop and sufficient labor force was available to work on the subsistence fields, African rice must have been grown extensively in New World countries with a substantial slave population originating from the rice–growing areas that stretched from Sierra Leone to Ghana (Brydon 1981; Richards 1996). Portères (1946, 1955, and 1960) provides evidence from botanical collections that *O. glaberrima* occurs in El Salvador and French Guiana, while Carney (2001 and 2005) presents historical records that strongly suggest that the crop was grown in Brazil, Jamaica, South Carolina, and Suriname. Today, however, the cereal is scarcely known outside its area of origin in West Africa. But even there, *O. glaberrima* is rapidly being replaced by *O. sativa* as a result of the long and frequent droughts in the last few decades and the consequent introduction of fast–growing Asian varieties by agricultural development projects (Linares 2002; Richards 1996).

According to Carney (2005), the most recent botanical voucher of *O. glaberrima* in South America was collected in 1938 around French Guiana’s capital city Cayenne by the French botanist A. Vaillant. It was cultivated by Maroons, descendants from enslaved Africans that escaped from plantations in the 17th and 18th centuries. After fighting for over a century for freedom from plantation slavery and independence from the Dutch colonial rule, Maroons managed to establish viable, autonomous communities in the dense tropical rainforests of Suriname. The Maroons, or Bush Negroes are they were called in the past, are divided into six “tribes” (Kwinti, Aucans, Saramaccans, Boni, Paramaccans, and Matawais), each with a different language and culture. Due to the scarce influence of Christianity, Maroon culture and religion are often considered the most “African” of the Americas (Herskovits and Herskovits 1934; Price 1996). After surviving in relative isolation for hundreds of years, Maroons now form Suriname’s third largest ethnic group. Despite their recent migration to Suriname’s capital Paramaribo, French Guiana, and the Netherlands, most Maroons continue to live in traditional forest communities in Suriname (Price 2002; St-Hilaire 2000). Homegrown, rain–fed rice still is an important staple food in Maroon communities (Fleury 1993; Hurault 1965; Price 1993). Just like their Jamaican counterparts (Bilby 2005), Maroons are also regarded as the specialists in herbal medicine and ritual knowledge in the Guianas (Price 2008). Currently, Maroons are the main harvesters, traders, and consumers of herbal medicine in Suriname (van Andel et al. 2007).

Not long after their escape, Maroons started to grow rice as a staple food around their hidden settlements. Mercenaries who were sent to capture the runaways encountered extensive rice fields in cleared swamps surrounding the temporary rebel camps (Stedman 1988: 417). Maroons interviewed by Hurault (1965) and Price (1983) claimed that rice originally came from Africa and that it was introduced to the New World and later taken to the forest camps by a female ancestor who smuggled the seeds in her hair. There are strong indications that Maroons have continued to cultivate African rice until today, long after *O. sativa* was established as the country’s main cash crop on Suriname’s tidal plains by Asian contract laborers in the 1930s (Ostendorf 1962). Each of the few studies on Maroon agriculture (Fleury 1993; Geijskes 1954; Hurault 1965; Price 1991; Renoux et al. 2003) mentions the cultivation of “red” or “wild” or “black rice.” After men have cleared and burned the fields, sowing, harvesting, and preparing the rice are mainly women’s tasks (Price 1993). Geijskes (1954) listed 21 local rice varieties grown by Paramaccan and Aucan Maroons along the Marowijne River. Hurault numbered a dozen varieties planted by the Aucan and Boni Maroons of French Guiana. Anthropologists Richard and Sally Price recorded names in the Saramaccan Maroon language for no less than 74 varieties of rice, including a “true red rice” and a “forest rice” or “wild rice” (mátu alísi in the Saramaccan language) that was used mainly in rituals (Price 1993). At that time it was unclear to them whether 18th–century Saramaccans cultivated this “wild rice” or simply gathered it in nearby forest swamps (Price 1991: 110). Unfortunately, none of these scholars ever collected a voucher of any of these rice cultivars. In this paper, I will explain how “wild rice” and “forest rice” are related to *O. glaberrima* and present evidence that this “lost crop of the enslaved Africans” is still cultivated in Suriname today.

## Methods

**Fig. 1:**
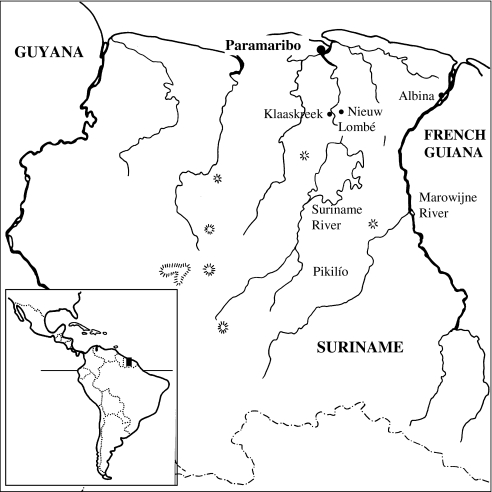
Map of Suriname, based on drawing by H. Rypkema.

Data on the cultivation and use of African rice were collected in the framework of the research project “Medicinal Plants of Suriname: Changes in Plant Use after Migration to the Netherlands.” Fieldwork took place from January to July 2006 and consisted of general ethnobotanical inventories, market surveys, and interviews around Paramaribo and along the lower Marowijne River (among the Aucan Maroons) and lower Suriname River (among Saramaccan Maroons) (Fig. 1). Additional rice samples were collected in Suriname in June 2008 and December 2009. A market survey among Surinamese herb shops in the Netherlands took place in October and November 2006 and additional data were gathered in January 2010. Specimens were collected of plants identified by informants as “wild rice,” “dog rice,” “forest rice,” or “black rice,” and rice used for ancestor offerings. Botanical collections of *Oryza* from Suriname were identified by B. Teeken and E. Nuijten of the Technology and Agrarian Development Group, Wageningen University. Vouchers were deposited at the National Herbarium of Suriname (BBS), the National Herbarium of the Netherlands (NHN–L), and the New York Botanical Garden (NYBG). Three samples of loose *Oryza* spikelets were deposited at the Economic Botany collection of the NHN–L. Fresh samples, purchased at the Paramaribo market in December 2009, were handed over to the Amsterdam Botanic Garden for planting trails and sent to Susan McCouch, Cornell University, for molecular analysis.

## Results

### Searching for African Rice in Herbaria and Floras

More than 20 species exist in the genus *Oryza* worldwide, of which only *O. sativa* and *O. glaberrima* are cultivated. Three wild species are listed for the Guianas: *O. grandiglumis* (Döll) Prodoehl, *O. latifolia* Desv., and *O. rufipogon* Griff (Judziewicz 1991). The latter species is a naturalized “rice weed” of Asian origin that has exchanged genes with *O. sativa* in the past and is still harvested by traditional communities in India (Vaughan et al. 2008). Prior to the research presented here, no vouchers of *O. glaberrima* were present in the Herbaria in Guyana (BRG), Suriname (BBS), or French Guiana (CAY), nor in the large Neotropical collections of the National Herbarium of the Netherlands (L), the New York Botanical Garden (NYBG), the Smithsonian Institution (US), and the Missouri Botanical Garden Herbarium (MBG). The specimen of *O. glaberrima* collected in 1938 by A. Vaillant (No. 24) is located in the in the Muséum National d’Histoire Naturelle (P) in Paris. Based on the observations of Portères (1955), *O*. *glaberrima* is included in the inventory of cultivated and adventive plants of French Guiana’s gardens (Hoff and Cremers 2005). However, *O. glaberrima* is not mentioned in the Flora of Suriname (Amshoff and Henrard 1943; Lindeman and Görts-van Rijn 1968), in the volume on the Poaceae in the Flora of the Guianas Series (Judziewicz 1991), or in the Checklist of the Plants of the Guiana Shield (Funk et al. 2007).

### African Rice and Its Use in Rituals

In 2006, small bags (ca. 60 gr.) of unmilled *O. glaberrima* and bags (120 gr.) of unmilled *O. sativa* spikelets were being sold by Maroon women for USD 1.30 each in the medicinal plant market in Paramaribo (van Andel et al. 2007). According to Aucan farmer Norbert Eersteling, during a ritual called “nyannyan mofu nayan,” small amounts of African rice are offered with some taro tubers (*Colocasia esculenta* [L.] Schott), a piece of sugarcane, and a few green bananas to the Earth Mother. This offering, known among Creoles as “ala mofo nyan” (literally “food for all mouths,” meaning an offering for all ancestors or gods), consists of a plate with boiled eggs, banana pudding, molasses, melegueta pepper (*Aframomum melegueta* [Roscoe] K. Schum.), maize, bananas, plantains, taro, and rice. Apparently, both African and Asian rice species figure in these ancestor offerings. The Boni Maroons serve dozens of different rice dishes during the ceremonies that mark the end of a mourning period (Fleury 1993).

The vendors of a Saramaccan herb shop and an Aucan market stall in Amsterdam said they occasionally sold “busi aleisi” or “blaka aleisi.” Unfortunately, the African rice was not in stock when we conducted our market surveys in 2000 (van Andel and van’t Klooster 2007) and in 2006 (Behari-Ramdas 2007). Unmilled Asian rice (*O. sativa*) was sold in both Maroon and East Indian shops in the Netherlands as it is offered during Maroon and Hindu ceremonies. In January 2010, a bag of loose, unmilled *O. glaberrima* spikelets was purchased in a Saramaccan “culture shop” in the East of Amsterdam. According to the shopkeeper, black rice was much tastier than normal rice, but it was rather rare and he had to order it all the way from his relatives in Klaaskreek. He sold bags of 50 gr for USD 6.00.

When I asked renowned Saramaccan tree spotter Frits van Troon about the origin of African rice, he said it was a wild plant that his ancestors had found growing on the edge of a swamp in the middle of the forest. They collected the panicles from this “natural rice field” and took them to their own gardens to plant the seeds. It was a rice species that matured in three months which, according to van Troon, “was handy for the Bush Negroes because they needed food quickly. They had little time to wait, since they had to escape further in the forest.” Van Troon remembered that his now–deceased mother still planted this fast–maturing variety of African cereal. Although he maintained the wild origin of the crop, reflected in its name “mátu alísi” (forest rice), he denied that it had anything to do with the wild grass known as “dagu aleisi” (dog rice, *Olyra latifolia* L.) that grows abundantly at forest clearings in Suriname.

### Finding Specimens

Table 1 lists all specimens identified by Maroons as “rice,” collected by the author in Suriname and the Netherlands in the period 2006–2008. It shows that not all grasses that Surinamers call “rice” belong to the genus *Oryza*, and not all “forest” or “bush” rice is *O. glaberrima*. Inedible species like *Oryza latifolia*, O. *rufipogon*, and *Olyra latifolia* are also called “wild,” “bush,” or “savannah rice” (Judziewicz 1991). Furthermore, unprocessed rice grains of both *O. glaberrima* and *O. sativa* figure in ancestor rituals.

**Table 1:** Collections of “rice” specimens from suriname and the netherlands made by the author

Species (Voucher)	Collection locality	Local name (language)	Use
*Oryza glaberrima* Steud. (TvA 5287)	Medicinal plant market, Paramaribo, Suriname	blaka aleisi, busi aleisi (Sr)**	ritual offering
*Olyra latifolia* L. (TvA 5523*)	Rijsdijkweg, Pará, Suriname	dagu aleisi (Sr) (dog rice)	ritual bath for babies, grains not edible
*Oryza sativa* L. (TvA 5583)	Aucan herb stall, Amsterdam	padi aleisi (Sr)	ritual offering
*Oryza glaberrima* Steud. (MJ 7075)	Nieuw Lombé, Suriname	baáka alísi, mátu alísi (Sar)	food
*Oryza glaberrima* Steud. (Tva 5632)	Nieuw Lombé, Suriname	baáka alísi, mátu alísi (Sar)	sowing material, food, ritual offering
*Oryza glaberrima* Steud. (TvA 5636)	Medicinal plant market, Paramaribo, Suriname	blaka aleisi, busi aleisi (Sr)	ritual offering
*Oryza glaberrima* Steud. (TvA 5637)	Saramaccan culture shop, Amsterdam.	blaka aleisi (Sr)	ritual offering

*Primary collector names: TVA = T.R. van Andel, MJ = M.J. Jansen-Jacobs.

**Sr Sranantongo, *Sar* Saramaccan Maroon language.

**Fig. 2:**
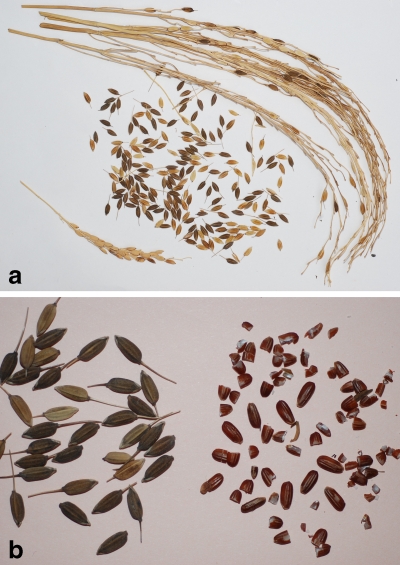
**a** Panicle of *Oryza glaberrima* kept as sowing material, Nieuw Lombé, Suriname (TvA 5632). The small panicle on the left hand corner is *O. sativa*. Photo: C. A. van der Hoeven. **b** Unmilled *O. glaberrima* spikelets, showing apical awn, and milled grains with a clear reddish–brown bran, both from the same sample (TvA 5635). Photo: C. A. van der Hoeven.

In 2008, after one of my samples was identified as *Oryza glaberrima*, I began looking for living specimens in the Saramaccan village of Nieuw Lombé (5°10′N, 55°04′W), where I had been doing fieldwork in 2006 (but never saw any field of African rice). Soon after I asked some villagers about the crop, they located a bag of uncleaned grains stored as sowing material, which consisted of an infrutescence with many loose seeds (Fig. 2a). The sowing material was adulterated with a small panicle of *O. sativa*. Figure 2b shows *O. glaberrima* grains with their husks still attached and milled rice grains, both from a sample bought at the Paramaribo market.

**Fig. 3:**
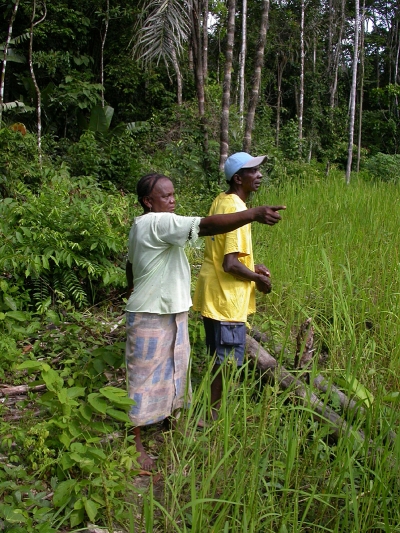
Emelina Saabo with Albie Poeketie in her rice field. Photo: M. J. Jansen–Jacobs.

**Fig. 4:**
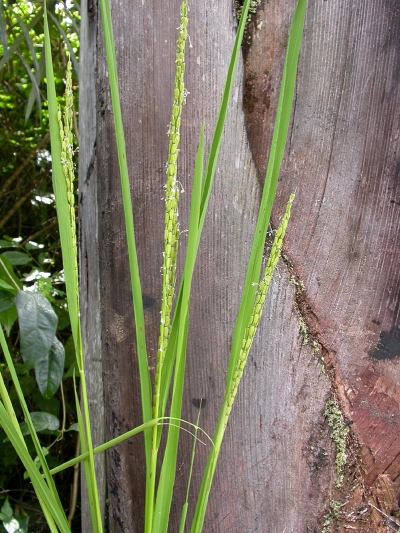
Flowering stalk of *Oryza glaberrima* (MJ 7075). Photo: M. J. Jansen–Jacobs.

The owner of the sowing material, a Saramaccan woman in her sixties named Emelina Saabo, confessed she had a whole field full of African rice (Fig. 3). She was reluctant to show her field to us, which was located a few kilometers from the village. Several “obias” (protective charms to ward off outsiders) were hanging along the entrance path to her garden. Emelina’s rice field measured some 30 m × 4 m, and the crop was in full flower (Fig. 4). Although the garden also contained cassava, taro, and tobacco, the rice was not intercropped but rather grown in a separate patch.

My field assistant, Saramaccan farmer Albie Poeketie, was not aware that his neighbor cultivated African rice and asked for some seeds to sow in his own garden. He told me that the species was more frequently cultivated in the past, but after a mechanical rice mill became operational in the neighboring village of Klaaskreek, farmers had shifted to “kuli alísi” (literally: “coolie rice”), the commercial Asian varieties grown by East Indians along the coast. He explained how African rice could only be milled by “máta ku tatí” (mortar and pestle), and afterwards needs to be winnowed by hand (“waai alísi”) in the large, richly decorated trays made from the buttresses of *Aspidosperma* trees (Apocynaceae). According to Poeketie, people found it too tiresome to pound off the rice husks by hand, even though many households in the village still owned wooden mortars.

When asked about the origin of African rice, Poeketie said he had never heard of the story of the Saramaccan woman who hid seeds in her hair. He remembered being told that his forefathers once found a large swamp in a forest where this type of rice was growing abundantly. All present–day African rice originated from the seeds his ancestors had collected from that field. He was sure that the mysterious rice swamp was not made by Maroons (or other human beings), since it was found it in an uninhabited stretch of forest.

## Discussion

### A Forest Spirit’s Rice Field

“Around 1800, one day when hunting on the Upper Pikilío, near Kwaminangoto, Gbagidi discovered a mysterious swamp surrounded by tempting bananas, wild rice, and various other crops. After cutting samples and setting out for home, he was horrified to see his favorite hunting dog being swallowed up by the swamp’s quicksand” (Price 1990: 250). This legend tells how a Saramaccan hunter accidentally disturbed the garden of an extraordinarily powerful “apuku” forest spirit. It bears a remarkable resemblance to the story my Saramaccan informants told, independently from each other, on how their ancestors discovered *Oryza glaberrima*. The fact that the rice field was made by a spirit of the deep woods may have led to the name “mátu alísi” (forest rice) and the strong claim that the plant was growing wild before Saramaccans started to cultivate it. How this mysterious rice swamp ever came to be, which rice species (*O. glaberrima*, *O. rufipogon*, or a hybrid?) was growing there, and whether it was related to the legend of the woman who hid the rice in her hair, we will probably never know. The importance, however, of Maroon oral history and ritual practices in the conservation of the different rice cultivars is evident.

### Traditional Religion, Development, and Agricultural Diversity

In many agricultural societies in the developing world, ancient food cultivars are grown for ceremonies to honor the link between crops and the ancestors. Sierra Leone farmers see rice genetic resources as an “ancestral blessing” (Richards 1996), while complex rituals surround the cultivation of *O. glaberrima* in Eastern Ghana (Brydon 1981). Senegalese farmers that were converted to Islam have replaced their crops with *O. sativa*, while those that maintained their traditional religion continued to plant African rice to honor their supreme deity, the rain god that gave *O. glaberrima* to their forbearers (Linares 2002). In Suriname, the Maroon culture is losing ground to the migration of their youth to the capital and evangelical pressure (St-Hilaire 2000). When people are converted to Christianity and refrain from offering “first–time” food to their ancestors and deities, the cultivation of this ancient landrace may be lost within one generation. At only a three–hour drive to the capital, Nieuw Lombé is a rather acculturated Maroon village, where several different Christian churches compete with the traditional Afro–Surinamese religion. African rice is still grown here, but it is done rather secretly and not openly shared with neighbors or outsiders.

Modern agricultural technology and the introduction of new high–yielding varieties are largely eliminating the wide range of crop genetic diversity that has evolved during the 5,000 to 10,000 years since food plants were first domesticated (Hawkes 2008). African rice may have a lower yield, but its pest resistance and adaptation to environmental stress perfectly suits the low–input agricultural system of the Maroons on the acid rainforest soils of Suriname. Moreover, it is likely that Maroons grow several cultivars of *O. glaberrima*, representing a genetic diversity that might differ from that in West Africa. Part of this genetic diversity may be caused by introgression of genetic material with *O. sativa* or the wild *O. rufipogon*, as is the case with *O. glaberrima* cultivated in Africa (Semon et al. 2004; Vaughan et al. 2008). Since domestication is a long–term process rather than a single event, some genetic diversity of the Surinamese varieties of *O. glaberrima* may have developed *after* their transatlantic journey.

Genetic resources of ancient rice cultivars are much needed in the creation of new crop varieties by plant breeders (Sarla and Mallikarjuna Swamy 2005; Vaughan et al. 2008). Successful cross experiments between *O. glaberrima* and *O. sativa* have led to new rice varieties named NERICA (“New Rice for Africa”) that combine the hardiness of the African species with the productivity of the Asian species (Linares 2002). Knowledge of the genetics of *O. glaberrima*, however, is far less than *O. sativa* and traditional landraces have been poorly collected prior to the introduction of improved varieties (Vaughan et al. 2008). Therefore, there is a great need for *in–situ* conservation programs of *O. glaberrima* on both sides of the Atlantic.

“Little research has focused on the role of provision gardens as the botanical gardens of the dispossessed, the marginal, those who struggled to hold on to their cultural identity under dehumanized conditions” (Carney 2001: 156). Carney’s plea may sound dramatic, but in the same village where we found the African rice, a large EU–funded development project was underway with the aim of integrating Maroon knowledge with modern agricultural techniques to develop sustainable agroforestry systems. Although an inventory of crops planted by local farmers was part of the project (Jorritsma 2006), no reference to African rice (or any other specific crop cultivar) was made in any of their reports (see www.guyagrofor.alterra.nl). A missed opportunity, since there are indications that additional “lost African crops” are still grown by Maroons or survive as relicts around former settlements, like the Bambara groundnut, *Vigna subterranea* (L.) Verdc. and the Senegal date palm, *Phoenix reclinata* Jacq. (van Andel et al. forthcoming; van Andel and van’t Klooster 2007; Price 1991).

## Conclusion

We can conclude that in the discussion of the survival of African cultural traditions among Afro–Americans (Mintz and Price 1992), the ongoing cultivation of African rice by Maroons in Suriname proves that more African traditions have survived than previously thought. At this moment, *Oryza glaberrima* is still grown, milled by hand, eaten, offered to the ancestors, sold on the market, and even exported to the Netherlands by Maroons. Traditional religion plays an important role in the survival of this ancient rice species, and Maroon women earn additional income by selling African rice seeds still in the husk for religious purposes. Previous research suggests that various cultivars of the cereal are grown, which may shed new light on the domestication process of African rice outside its center of origin. Much of the historical rice diversity has likely been lost before scientists were able make collections and store germplasm in gene banks. Therefore, it is imperative to collect and describe the existing rice cultivars grown by the different Maroon tribes in Suriname before they disappear through the introduction of improved varieties, shortage of labor due to migration, or loss of traditional religion. Further research on Maroon agriculture may also reveal more “lost crops” that have disappeared from the former plantations, but are still cherished by the descendants of those who fled them.

## References

[CR1] Amshoff, G. J. H. and J. T. Henrard. 1943. Gramineae. Pages 273–442 in A. A. Pulle, ed., Flora of Suriname 1(1), Utrecht, the Netherlands.

[CR2] Behari-Ramdas, J. A. 2007. Evaluating Ecological Impact of Commercial Trade on Surinamese Medicinal Plants Sold in the Netherlands. M.Sc. Thesis, Department of Science, National Herbarium of the Netherlands, Leiden University. http://osodresie.wikispaces.com/Student%27s+thesis (5 January 2010).

[CR3] Bezançon, G. and S. Diallo. 2006. *Oryza glaberrima* Steud. Record from Protabase. M. Brink and G. Belay, eds., PROTA (Plant Resources of Tropical Africa), Wageningen, the Netherlands. http://database.prota.org/search.htm (22 September 2009).

[CR4] Bilby K (2005). True–Born Maroons.

[CR5] Brydon L (1981). Rice, Yams and Chiefs in Avatime: Speculations on the Development of a Social Order. Africa.

[CR6] Carney JA (2001). Black Rice: The African Origins of Rice Cultivation in the Americas.

[CR7] Carney JA (2005). Rice and Memory in the Age of Enslavement: Atlantic Passages to Suriname. Slavery and Abolition.

[CR8] Fleury M, Hladik CM, Hladik A, Linares OF, Pagezy H, Semple A, Hadley M (1993). Food Plants and Cultural Identity: The Boni in French Guiana and African Memories. Tropical Forests, People and Food. Man and the Biosphere Series 13.

[CR9] Funk V, Hollowell T, Berry P, Kellof C, Alexander SN (2007). Checklist of the Plants of the Guiana Shield.

[CR10] Geijskes DC (1954). De landbouw bij de Bosnegers van de Marowijne. West–Indische Gids.

[CR11] Harlan J (1995). The Living Fields: Our Agricultural Heritage.

[CR12] Hawkes JG (2008). The Importance of Genetic Resources in Plant Breeding. Biological Journal of the Linnean Society.

[CR13] Herskovits MJ, Herskovits FS (1934). Rebel Destiny: Among the Bush Negroes of Dutch Guiana.

[CR14] Hoff M, Cremers G (2005). Le Jardin Guyanais: Inventaire des plantes cultivées et des adventices des jardins de Guyane française. Journal Botanique de la Société Botanique de France.

[CR15] Hurault J (1965). La Vie Matérielle des Noirs Réfugiés Boni et des Indiens Wayana du Haut–Maroni, Guyane Française.

[CR16] Jorritsma, F. 2006. Understanding Soil Fertility Management of Maroon Farmers. M.Sc. thesis, Land Degradation and Development Group, Wageningen University, Wageningen, the Netherlands.

[CR17] Judziewicz EJ, Görts-van Rijn ARA (1991). Flora of the Guianas 8.

[CR18] Linares OF (2002). African Rice (*Oryza glaberrima*): History and Future Potential. Proceedings of the National Academy of Sciences.

[CR19] Lindeman, J. C. and A. R. A. Görts-van Rijn. 1968. Graminae. Pages 343–375 in A. A. Pulle and J. Lanjouw, eds., Flora of Suriname 1(2), Utrecht, the Netherlands.

[CR20] Mintz SW, Price R (1992). The Birth of an African–American Culture: An Anthropological Approach.

[CR21] Ostendorf, F. W. 1962. Nuttige planten en sierplanten in Suriname. Landbouwproefstation in Suriname, Paramaribo, Suriname.

[CR22] Portères R (1946). Systematique intraspecifique chez *Oryza glaberrima*. Revue International de Botanique Appliquée et d’Agriculture Tropicale.

[CR23] Portères R (1955). Présence ancienne d’une variété cultivée d’*Oryza glaberrima* St. en Guyane Française. Journal d’Agriculture Tropicale et de Botanique Appliquée.

[CR24] Portères R (1960). Riz subspontanés et riz sauvages en El Salvador (Amérique Centrale). Journal d’Agriculture Tropicale et de Botanique Appliquée.

[CR25] Price R (1983). First–Time: The Historical Vision of an Afro–American People.

[CR26] Price R (1990). Alabi’s World.

[CR27] Price R (1991). Subsistence on the Plantation Periphery: Crops, Cooking, and Labour among Eighteenth–Century Suriname Maroons. Slavery and Abolition.

[CR28] Price R (1996). Maroon Societies: Rebel Slave Communities in the Americas.

[CR29] Price R (2002). Maroons in Suriname and Guyane: How Many and Where. New West Indian Guide.

[CR30] Price R (2008). Travels with Tooy: History, Memory, and the African American Imagination.

[CR31] Price S (1993). Co–wives and Calabashes.

[CR32] Renoux F, Fleury M, Reinette Y, Grénand P, Grénand F (2003). L’ágriculture itinérante sur brûlis dans les bassins du Maroni et de l’Oyapock: dynamique et adaptation aux contraintes spatiales. Revue Forestière Française.

[CR33] Richards P, Brush S, Stablinsky D (1996). Culture and Community Values in the Selection and Maintenance of African Rice. Indigenous People and Intellectual Property Rights.

[CR34] Salley AS (1919). Introduction of Rice into South Carolina. Bulletins of the Historical Commission of South Carolina.

[CR35] Sarla N, Mallikarjuna Swamy BP (2005). *Oryza glaberrima*: A Source for the Improvement of *Oryza sativa*. Current Science.

[CR36] Semon M, Nielsen R, Jones MP, McCouch SR (2004). The Population Structure of African Cultivated Rice *Oryza glaberrima* (Steud.): Evidence for Elevated Levels of Linkage Disequilibrium Caused by Admixture with *O. sativa* and Ecological Adaptation. Genetics.

[CR37] St-Hilaire A (2000). Global Incorporation and Cultural Survival: The Surinamese Maroons at the Margins of the World–System. Journal of World–Systems Research.

[CR38] Stedman, J. G. 1988. Narrative of a Five Years’ Expedition against the Revolted Negroes of Surinam. Transcribed for the first time from the original 1790 manuscript by R. Price and S. Price, eds., The Johns Hopkins University Press, Baltimore, Maryland.

[CR39] van Andel TR, Van’t Klooster CIEA, Pieroni A, Vandebroek I (2007). Medicinal Plant Use by Surinamese Immigrants in Amsterdam, the Netherlands. Traveling Cultures and Plants and Medicines: The Ethnobiology and Ethnopharmacy of Human Migrations.

[CR40] van Andel TR, Behari-Ramdas JA, Havinga RM, Groenendijk S (2007). The Medicinal Plant Trade in Suriname. Ethnobotany Research and Applications.

[CR41] van Andel TR, Maas PJM, Dobreff J, Dobreff J (2010). Are Rolander’s plants still used in Suriname today?. The Annotated Version of Daniel Rolander’s Diarium Surinamicum.

[CR42] Vaughan DA, Lu B, Tomooka N (2008). The Evolving Story of Rice Evolution. Plant Science.

